# Supplementary material for: A critical re-evaluation of fMRI signatures of motor sequence learning
Source: eLife. 2020 May 13;9:e55241. doi: 10.7554/eLife.55241 (PMC7266617; doi:10.7554/eLife.55241)
Supplement: Transparent reporting form [file elife-55241-transrepform.docx]

***eLife’s* transparent reporting form**

We encourage authors to provide detailed information *within their submission* to facilitate the interpretation and replication of experiments. Authors can upload supporting documentation to indicate the use of appropriate reporting guidelines for health-related research (see [EQUATOR Network](http://www.equator-network.org/%20)), life science research (see the [BioSharing Information Resource](https://biosharing.org/)), or the [ARRIVE guidelines](http://www.plosbiology.org/article/info:doi/10.1371/journal.pbio.1000412) for reporting work involving animal research. Where applicable, authors should refer to any relevant reporting standards documents in this form.

If you have any questions, please consult our Journal Policies and/or contact us: [editorial@elifesciences.org](mailto:editorial@elifesciences.org).

**Sample-size estimation**

- You should state whether an appropriate sample size was computed when the study was being designed
- You should state the statistical method of sample size computation and any required assumptions
- If no explicit power analysis was used, you should describe how you decided what sample (replicate) size (number) to use

Please outline where this information can be found within the submission (e.g., sections or figure legends), or explain why this information doesn’t apply to your submission:

Given the range of questions we wanted to address, we did not use a formal power analysis to determine sample size. Based on prior studies, a sample size of at least N=20-30 was judged to be adequate to test the main hypotheses. We therefore pre-registered a sample size of N=25, and ended up including 26 subjects. The sample size can be found in the section ‘Participants’ in Materials and Methods.

**Replicates**

- You should report how often each experiment was performed
- You should include a definition of biological versus technical replication
- The data obtained should be provided and sufficient information should be provided to indicate the number of independent biological and/or technical replicates
- If you encountered any outliers, you should describe how these were handled
- Criteria for exclusion/inclusion of data should be clearly stated
- High-throughput sequence data should be uploaded before submission, with a private link for reviewers provided (these are available from both GEO and ArrayExpress)

Please outline where this information can be found within the submission (e.g., sections or figure legends), or explain why this information doesn’t apply to your submission:

The details about the paradigm (how often experiment was performed in the scanner, and how many behavioral sessions were included) can be found in the Materials and Methods section – specifically the ‘Learning paradigm’ and ‘Experimental design during scanning’ subsections. The inclusion criteria (handedness, no neurological disorders) are specified under the ‘Participants’ section in the Methods. The distinction between biological and technical replicates, and sequence data do not apply to this submission.

**Statistical reporting**

- Statistical analysis methods should be described and justified
- Raw data should be presented in figures whenever informative to do so (typically when N per group is less than 10)
- For each experiment, you should identify the statistical tests used, exact values of N, definitions of center, methods of multiple test correction, and dispersion and precision measures (e.g., mean, median, SD, SEM, confidence intervals; and, for the major substantive results, a measure of effect size (e.g., Pearson's r, Cohen's d)
- Report exact p-values wherever possible alongside the summary statistics and 95% confidence intervals. These should be reported for all key questions and not only when the p-value is less than 0.05.

Please outline where this information can be found within the submission (e.g., sections or figure legends), or explain why this information doesn’t apply to your submission:

Statistical analysis methods are described in the following sections: “Changes in overall activation”, “Dissimilarities between sequence-specific activity patterns” and “Pattern component analyses: modelling sequence-specific correlation across sessions”. For each performed statistical test, the exact t / F / p values are reported throughout the results section.

(For large datasets, or papers with a very large number of statistical tests, you may upload a single table file with tests, Ns, etc., with reference to sections in the manuscript.)

**Group allocation**

- Indicate how samples were allocated into experimental groups (in the case of clinical studies, please specify allocation to treatment method); if randomization was used, please also state if restricted randomization was applied
- Indicate if masking was used during group allocation, data collection and/or data analysis

Please outline where this information can be found within the submission (e.g., sections or figure legends), or explain why this information doesn’t apply to your submission:

Participants were randomly assigned to one of the two experimental groups (with different trained sequences). This is specified in the ‘Learning paradigm’ part of the Methods. The group assignment was not of importance to participants as the study design was still within-subject. Therefore, no blinding or masking was used.

**Additional data files (“source data”)**

- We encourage you to upload relevant additional data files, such as numerical data that are represented as a graph in a figure, or as a summary table
- Where provided, these should be in the most useful format, and they can be uploaded as “Source data” files linked to a main figure or table
- Include model definition files including the full list of parameters used
- Include code used for data analysis (e.g., R, MatLab)
- Avoid stating that data files are “available upon request”

Please indicate the figures or tables for which source data files have been provided:

The code for data analysis, together with source data to reproduce the main figures, is available on <https://github.com/eberlot/motor_sequence_learning>.
