# Supplementary material for: A critical re-evaluation of fMRI signatures of motor sequence learning
Source: eLife. 2020 May 13;9:e55241. doi: 10.7554/eLife.55241 (PMC7266617; doi:10.7554/eLife.55241)
Supplement: Peer review history

# Decision letter

**DOI:** 10.7554/eLife.55241.sa1

**Affiliations:** Radboud UniversityNetherlands; Moss Rehabilitation Research InstituteUnited States

In the interests of transparency, eLife publishes the most substantive revision requests and the accompanying author responses.

**Acceptance summary:**

This longitudinal fMRI study used a large sample, preregistered methods, and advanced analyses to test previous, sometimes conflicting, findings in the domain of motor sequence learning. The study provides convincing evidence for motor sequence learning outside of primary motor cortex. The work is a tour-de-force involving four scanning sessions and thousands of behavioural practice trials.

**Decision letter after peer review:**

Thank you for submitting your article "A critical re-evaluation of fMRI signatures of motor sequence learning" for consideration by *eLife*. Your article has been reviewed by three peer reviewers, one of whom is a member of our Board of Reviewing Editors, and the evaluation has been overseen by Floris de Lange as the Senior Editor. The following individual involved in review of your submission has agreed to reveal their identity: Aaron L Wong (Reviewer #2).

The reviewers have discussed the reviews with one another and the Reviewing Editor has drafted this decision to help you prepare a revised submission.

Summary:

This paper reports a longitudinal fMRI study investigating motor sequence learning. The study aimed to verify previous (sometimes conflicting) findings using a relatively large sample and preregistered methods. The study also tested new hypotheses and directly compared speeded and paced performance of trained and untrained sequences.

The authors find that, consistent with their previous work, they see no evidence of sequence-specific changes in M1; in association cortices, they observe average activity decreases associated with sequence learning, with sequence-specific activity patterns changing primarily early in learning.

All reviewers were impressed with the quality of the design and analysis of the study, and agreed that it will provide an important contribution to the literature. The work is a tour-de-force involving four scanning sessions and thousands of behavioral practice trials. It is great, therefore, that this valuable dataset will be made publicly available, such that other researchers can test their own hypotheses as well.

Essential revisions:

1) While the authors have carefully controlled for movement speed, it isn't clear whether they also took into consideration two other related factors that likely affect performance and neural activity: the error rate, and the amount of reward (i.e., points) earned. Given that MT in the first test session is typically faster than during initial training, individuals seem likely to make a fair number of errors in Week 1 that would largely decrease with training (since MT in week 2 is now faster in training than during test, it seems likely that the largest change in error/reward rate occurs between Weeks 1-2). It is important to know what performance accuracy was like across weeks, if incorrect trials are excluded from the fMRI analysis, and whether error/reward rate might have contributed to changes in neural activity (e.g., in the striatum).

2) On a related note, it is strange that individuals were performing nearly 1 second faster during Test 1 compared to the start of training (i.e., Figure 2 comparing panel C and D) despite a lack of pacing in both cases. Was this slowdown systematic across individuals, and what would have caused it? Likewise, it is curious that MTs in the initial test session is so much faster than the initial training session (but in following weeks MT is on par or slower in the testing session compared to training). This seems to call into question the accuracy of the baseline measurements taken in Week 1 prior to training. It would be helpful for the authors to comment on this.

3) Finally, it is curious that some patterns of activity only become evident at full speed (e.g., in the striatum). Does this mean that, akin to a model where neural activity scales with movement speed, moving faster simply pulls the same signal out of the noise better (which seems to be true in cortex)? Or does this imply that moving at peak speed actually changes the patterns of neural activity in some way? The authors address this question for cortex (subsection “Trained sequences elicit distinct patterns during full speed performance”, third paragraph), but should perform a similar analysis for the striatum to know how similar sequence representation patterns are throughout the brain. This would also speak to the authors' discussion of the role of the striatum in motor sequence learning.

# Reply: Author response

**DOI:** 10.7554/eLife.55241.sa2

Essential revisions:

1) While the authors have carefully controlled for movement speed, it isn't clear whether they also took into consideration two other related factors that likely affect performance and neural activity: the error rate, and the amount of reward (i.e., points) earned. Given that MT in the first test session is typically faster than during initial training, individuals seem likely to make a fair number of errors in Week 1 that would largely decrease with training (since MT in week 2 is now faster in training than during test, it seems likely that the largest change in error/reward rate occurs between Weeks 1-2). It is important to know what performance accuracy was like across weeks, if incorrect trials are excluded from the fMRI analysis, and whether error/reward rate might have contributed to changes in neural activity (e.g., in the striatum).

Thank you for raising this point. For the fMRI analysis presented in the original submission, we did not exclude errors trials, which has been clarified in text now. This is an approach we’ve commonly taken in our previous papers (e.g. Yokoi and Diedrichsen, 2019; Yokoi et al., 2018; Ejaz et al., 2015, etc.), as the dominant form of error in this paradigm are small execution errors (i.e., finger failed to reach force threshold), such that most of the normal planning- and execution related activity is likely preserved. Exclusion of errors introduces an unequal trial number across conditions and runs, which leads to decreased power and can also introduce biases into the multivariate analysis. However, it is a serious concern and we therefore have conducted a range of additional analyses to confirm that errors did not affect our cross-session correlation analyses.

First off, the reward rate inside the scanner was purely dependent on correctness of execution – i.e. if inside the scanner participants performed the sequence correctly, they received 3 points, and 0 otherwise. This differs from the approach we took for reward system in training sessions, where the additional criterion was the speed of performance, to keep participants motivated throughout learning. Since scans 1-3 were performed at a paced speed, we omitted this speed-dependence on reward in all scanning sessions. This also prevented a difference in reward / points given between paced and full speed sessions.

During behavioral training, participants were instructed to go as fast as possible, while keeping the error rate below 15%. Errors were more frequent in the first two sessions (which was mainly due to the second trial where they had to perform sequences from memory – see response to the 2^nd^ point), but overall hovered around 15%, as shown in Author response image 1A. As in behavioral sessions, participants were told to keep their error rate below 15% also in scanning sessions. However, the number of errors executed varied significantly across sessions, and between sequence types (see Author response image 1B). A session x sequence type ANOVA was significant for both session (*F*_(3,75)_=9.21, *p*=2.93e^-5^) and sequence type (*F*_(1,25)_=11.15, *p*=2.64e^-3^). There was also a significant interaction (*F*_(3,75)_=8.42, *p*=6.75e^-5^). Post-hoc t-tests revealed that the error rate differed between trained and untrained sequences in week 2 (*t*_(25)_=4.22, *p*=2.84e^-4^) and week 5 (*t*_(25)_=4.81, *p*=6.1e^-5^), but not in week 1, or in the speeded session of week 5 (5* in Author response image 1B). Comparing error rate across sessions, week 1 had overall more errors than week 2 and 5 (*t*_(25)_>=2.94, *p*<=0.007). Similarly, errors were more frequent in speeded session than the paced sessions (week 2 and 5) (*t*_(25)_>=2.40, *p*<=0.024). The speeded session and week 1, however, did not differ significantly in terms of number of errors.

**Author response image 1.** Error rate in behavioral and scanning sessions. a) Percentage of erroneous trials for trained sequences throughout behavioral training days. b) Percentage of errors for trained (red) and untrained (blue) sequences in the scanning sessions across weeks 1, 2 and 5 (1, 2, 5 at paced speed, 5* at full speed).

Since errors indeed differed between the paced session in week 1 and 2, and were different for trained than untrained sequences, the concern you are bringing is valid – it could be that differences in pattern correlation across sessions are due to differences in the number of errors across the two sessions. To addressed this concern, we completely re-did the fMRI analysis, starting with a first level general linear model that estimated the activity patterns for each sequence and run using only the correct trials. Error trials were modelled by a separate regressor that was not used in subsequent analyses. We then re-did the PCM correlation analysis on these newly estimated betas. The general pattern of observed correlation was very similar to the previous analysis (Author response image 2A – with error trials, Author response image 2B – without error trials).

To statistically quantify the results, we performed a crossvalidated t-test, as in the original submission. We used a one-sided threshold for significance since our hypothesis was directional, predicting that trained patterns would display a lower correlation across weeks 1-2 than untrained ones. As with the original analysis, we observed that trained patterns displayed significantly lower correlation across weeks 1 and 2 than untrained patterns in PMd (*t*_(25)_=1.72, *p*=.048) and SPLa (*t*_(25)_=1.71, *p*=0.049). This difference between trained and untrained patterns across weeks 1-2 was not significant for M1 (*t*_(25)_=0.87, *p*=0.40), or S1 (*t*_(25)_=0.53, *p*=0.15). Additionally, as reported previously, this difference in correlations between trained and untrained patterns was no longer present across weeks 2 and 5 (PMd: *t*_(25)_=0.08, *p*=0.46; SPLa: *t*_(25)_=0.96, *p*=.17). Although the significance of the effects is not as strong as with the errors included, the mean estimates of the most likely correlation did not change systematically when excluding errors. Therefore, we believe that this reduction in significance is caused mainly by the increased variability of the estimates caused be the inclusion of fewer trials into the analysis.

**Author response image 2.** Relative log-evidence (log marginal likelihood) for different correlation models across sessions (week 1-2 in solid lines, week 2-5 in dotted lines) for trained (red) and untrained (blue) activity patterns. Regions: PMd (dorsal premotor cortex), SPLa (superior parietal lobule – anterior part), M1 (primary motor cortex), S1 (primary somatosensory cortex). Difference between log-likelihoods can be interpreted as log-Bayes factor, with a difference of 1 indicating positive evidence. Vertical lines indicate the winning correlation model for trained (red) and untrained (blue) patterns across weeks 1-2. Black dots are projections of the two winning models onto the correlation function of trained sequences (weeks 1-2). The horizontal lines from the two black dots indicate the likelihood of the trained data under the two models, which was tested in a cross validated t-test. a) Evidence based on preprocessing, reported in manuscript (here just adjusted in y-axis. b) Evidence for correlations after removing error trials from a general linear model estimation.

We then systematically inspected the other reported results, to determine if they could have been affected by difference in error rate between trained and untrained sequences in the paced sessions in weeks 2 and 5. This observed pattern of errors could have contributed to differences in overall evoked activity in premotor and parietal areas. Namely, we observed reduced activation for trained sequences in weeks 2 and 5 (Author response image 3A), where also the number of errors was lower for trained sequences. Even after removing error trials, activation was still significantly lower in PMd and SPLa in weeks 2 and 5 for trained than untrained sequences (Author response image 3A – with error trials, Author response image 3B – without error trials), suggesting that the observed reduced activation was not due to lower error rates for trained sequences.

**Author response image 3.** Elicited activation, measured as percent signal change over resting baseline across weeks for trained (red) and untrained (blue) sequences. Regions: M1 (primary motor cortex), S1 (primary somatosensory cortex), PMd (dorsal premotor cortex), SPLa (superior parietal lobule – anterior part). a) Estimated elicited activation based on preprocessing, reported in manuscript. b) Estimated elicited activation after removing error trials from a general linear model estimation.

Similarly, the results concerning the cosine angle dissimilarity between average trained and untrained sequences were largely unaffected by difference in error rate in weeks 2 and 5 (Author response image 4) – for both PMd and SPLa the cosine angle increased significantly across weeks. The only difference was that previously significant increase in average cosine angle from week 1 to 2 in S1 was no longer significant (*t*_(25)_=1.97, *p*=0.060). Already in the original analysis the increase across weeks was smaller in S1 than in PMd and SPLa.

**Author response image 4.** Dissimilarity between average trained and untrained sequence, measured with cosine angle. Regions: M1 (primary motor cortex), S1 (primary somatosensory cortex), PMd (dorsal premotor cortex), SPLa (superior parietal lobule – anterior part). a) Estimated elicited activation based on preprocessing, reported in manuscript. b) Estimated elicited activation after removing error trials from a general linear model estimation.

Finally, since the error rate did not differ significantly between trained and untrained sequences the speeded session of week 5, our results of higher dissimilarities for trained than untrained sequences at full speed cannot be due to difference in error or reward. Similarly, in the striatum, we observed significant dissimilarities only for trained sequences in session 4, which thus cannot be driven by error/reward rate.

We have now clarified treatment of errors in our analysis pipeline in the manuscript.

Changes made:

“All instances of sequence execution were included into estimating regressors, regardless of whether the execution was correct or erroneous (see section Treatment of error trials below).”

“Treatment of error trials

As in behavioral sessions, participants were instructed to keep their error rate below 15% also inside the scanner. […] Indeed, our results did not differ qualitatively when excluding errors, therefore we here report only the analyses with all trials included.”

We have also added an explicit description of reward rate for experimental design during scanning.

Changes made:

“Participants always received 3 points upon correct execution of the sequence, and 0 points otherwise.”

Additionally, we have commented on the results of analyses without error trials in the main Results section, and referred the reader to the newly added Figure 3—figure supplement 1, and Figure 6—figure supplement 1A.

Changes made:

“This pattern of results on changes in overall activation remained unchanged after excluding error trials from the analyses (see Figure 3—figure supplement 1A).”

“This observed increase in dissimilarity between average trained and untrained pattern in PMd and SPLa, and to a lesser extent in S1, was also observed when analyzing only trials with correct performance (see Figure 3—figure supplement 1B).”

“To ensure that the observed lower correlation for trained patterns was not due to larger difference in error rate between weeks 1 and 2 for trained than for untrained sequences, we repeated the analysis excluding error trials. […] In S1, after accounting for error trials the correlation across weeks 1-2 did no longer differ between trained and untrained patterns.”

2) On a related note, it is strange that individuals were performing nearly 1 second faster during Test 1 compared to the start of training (i.e., Figure 2 comparing panel C and D) despite a lack of pacing in both cases. Was this slowdown systematic across individuals, and what would have caused it? Likewise, it is curious that MTs in the initial test session is so much faster than the initial training session (but in following weeks MT is on par or slower in the testing session compared to training). This seems to call into question the accuracy of the baseline measurements taken in Week 1 prior to training. It would be helpful for the authors to comment on this.

The main reason for the slowdown was the fact that in training, participants performed the sequences from memory. Specifically, each sequence presented twice: during the first execution, the full 9 digits were presented, whereas the second execution needed to be done from memory. We adopted this approach to encourage participants to form an explicit memory of the sequences, as we have done in several previous studies (Yokoi and Diedrichsen, 2019; Yokoi et al., 2018; Popp et al., 2019). This means that, especially early in learning, participants had to simultaneously memorize a 9-digit sequence while performing it for the first time, and then execute it from memory the second time around. In contrast, during testing and scanning sessions, where both trained and untrained sequences had to be performed, the full 9-digit sequences were always presented, thereby removing memory requirements. Thus, any comparison between training (Figure 2C) and testing (Figure 2D) needs to be made with caution.

The extra load to memorize the sequence slowed participants down substantially on the first day. Participants were significantly slower on the second, memorized, execution (Author response image 5A; *t*_(25)_=3.17, *p*=.004), and made more errors on the second execution (Author response image 5B). However, it is very likely that participants also slowed down the first execution to be able to better encode the sequence. Therefore, test session 1 better indicates the speed participants were able to perform at motorically. Already on training day 2, there was no difference in speed for first and second execution.

**Author response image 5.** Changes in movement time and error in behavioral training sessions, split between executions 1 and 2. a) Movement time to execute trained sequences across training days, split between execution 1 – digits presented on the screen, and execution 2 – from memory. b) Error rate, split between executions 1 and 2 for training days.

While our behavioral training sessions were designed to motivate participants to learn specific trained sequences, behavioral test sessions were in place to compare performance of trained to untrained sequences. Those two types of sessions therefore required different design choices. Importantly, none of our results or conclusions rely on the behavioral performance during training. We have now clarified some of this confusion in the main text.

“During the behavioral test sessions (Figure 2D), participants executed both the trained sequences, as well as matched untrained sequences, with all sequences randomly interspersed. […] Therefore, the requirement to remember the sequences from the first to second execution, which was present in training sessions, was omitted for test sessions. For this reason, performance in training and test sessions (Figure 2C-D) cannot be directly compared.”

3) Finally, it is curious that some patterns of activity only become evident at full speed (e.g., in the striatum). Does this mean that, akin to a model where neural activity scales with movement speed, moving faster simply pulls the same signal out of the noise better (which seems to be true in cortex)? Or does this imply that moving at peak speed actually changes the patterns of neural activity in some way? The authors address this question for cortex (subsection “Trained sequences elicit distinct patterns during full speed performance”, third paragraph), but should perform a similar analysis for the striatum to know how similar sequence representation patterns are throughout the brain. This would also speak to the authors' discussion of the role of the striatum in motor sequence learning.

This is a very interesting point. It could be that while in the cortical regions the patterns during full speed performance just become clearer, yet still qualitatively similar to those in the paced performance, the striatum shows a qualitative shift in responses from paced to full speed performance. As you are mentioning, our analysis of dissimilarities alludes to this possibility since sequence-specific striatal patterns were only significant for trained sequences during full speed execution. A correlation of sequence-specific patterns across the paced and full speed sessions in week 5 would allow us to address this question more directly. The results of the analysis are presented in Author response image 6. Similar to the regions in the cortex, there was some correlation of activity patterns across paced and full speed sessions – this correlation was clearly higher than 0 (putamen: *t*_(25)_=9.56, *p*=8.0e^-10^; caudate: *t*_(25)_=6.37, *p*=1.1e^-6^), indicating some consistency across different speeds. On the other hand the correlation was, even after accounting for measurement noise, lower than 1 (putamen: *t*_(25)_=8.85, *p*=3.6e^-9^; caudate: *t*_(25)_=5.86, *p*=4.1e^-6^), suggesting a modest change across speeds. Thus, although the correspondence wasn’t perfect, the results nevertheless suggests that, similarly to cortical regions, performance at full speed makes fine-grained changes in activity more pronounced, i.e. pulls the signal out of the noise.

Also similar to what we’ve observed in cortical regions, there was no significant evidence that the correlation differed between trained and untrained sequences. Specifically, the evidence of the trained patterns under the winning model for trained and untrained sequences (assessed in cross validated fashion) was not significant different – this was the case for both the putamen (*t*_(25)_=0.58, *p*=.57) and caudate (*t*_(25)_=1.43, *p*=.17). While based on the figure, there seems to be an interaction in the observed correlation shapes for trained and untrained sequences across the two regions, this was also not significant, as determined by a region x sequence type ANOVA (region: *F*_(1,25)_=0.19, *p*=.66; sequence type: *F*_(1,25)_=0.054, *p*=.83, interaction: *F*_(1,25)_=0.92, *p*=.35).

**Author response image 6.** Evidence for different correlation models, across paced and full speed sessions in week 5, for trained (red) and untrained (blue) patterns in putamen and caudate. Model evidence was measured as a log-Bayes factor relative to the average log-Bayes across all models (horizontal line at 0). The red and blue lines are drawn for correlation model with highest evidence for trained and untrained patterns, respectively. Vertical lines indicate winning model for trained and untrained sequences. Horizontal lines indicate the difference in log-likelihood of the trained data under these two model, which was evaluated in a cross validated fashion.

We have now added this analysis to our Results section on the striatal changes with learning.

Changes made in the Results section:

“To examine whether the speed purely pulls the signal out of the noise better, or qualitatively changes the representation, we, similarly to the analyses in the cortical regions, performed the PCM correlation model across the paced and full speed sessions in week 5. […] This suggests that moving faster engages similar representations as moving slower, but helps to increase the signal-to-noise ratio.”

Changes made in the Discussion:

“Additionally, our results suggest that full speed performance might be particularly important for further studies of striatal multivariate activation to counteract the generally lower signal-to-noise ratio in this region.”
